# Tuning the Weak Ferromagnetic States in Dysprosium Orthoferrite

**DOI:** 10.1038/srep37529

**Published:** 2016-11-25

**Authors:** Shixun Cao, Lei Chen, Weiyao Zhao, Kai Xu, Guohua Wang, Yali Yang, Baojuan Kang, Hongjian Zhao, Peng Chen, Alessandro Stroppa, Ren-Kui Zheng, Jincang Zhang, Wei Ren, Jorge Íñiguez, L. Bellaiche

**Affiliations:** 1 Department of Physics, and International Center of Quantum and Molecular Structures, Shanghai University, Shanghai 200444, China; 2 Materials Genome Institute, and Shanghai Key Laboratory of High Temperature Superconductors, Shanghai University, Shanghai 200444, China; 3 State Key Laboratory of High Performance Ceramics and Superfine Microstructure, Shanghai Institute of Ceramics, Chinese Academy of Sciences, Shanghai 200050, China; 4 Materials Research and Technology Department, Luxembourg Institute of Science and Technology (LIST), 5 avenue des Hauts-Fourneaux, L-4362 Esch/Alzette, Luxembourg; 5 CNR SPIN, Via Vetoio, I-67100 Laquila, Italy; 6 Physics Department and Institute for Nanoscience and Engineering, University of Arkansas, Fayetteville, AR 72701, USA

## Abstract

RFeO_3_ orthoferrites, where R is a rare-earth ion of the lanthanide series, are attracting attention mostly because of their promising fast spin dynamics. The magnetic properties of these materials seem to crucially depend on whether the magnetizations of the R and Fe ions’ weak ferromagnetic (WFM) components are parallel or antiparallel to each other. Here, we report an extensive investigation of a high-quality DyFeO_3_ single crystal in which the induced Dy^3+^ magnetization (F_Dy_) has a natural tendency to be antiparallel to Fe^3+^ sublattice magnetization (F_Fe_) within a large temperature window. Moreover, we find that specific variations of temperature and applied magnetic fields allow us to make F_Dy_ parallel to F_Fe_, or force a spin-flip transition in F_Fe_, among other effects. We found three different magnetic states that respond to temperature and magnetic fields, i.e. linear *versus* constant or, alternatively, presenting either behavior depending on the history of the sample. An original magnetic field-versus-temperature phase diagram is constructed to indicate the region of stability of the different magnetic phases, and to reveal the precise conditions yielding sudden spin switching and reversals. Knowledge of such a phase diagram is of potential importance to applications in spintronics and magnetic devices.

Dysprosium orthoferrite DyFeO_3_ (DFO) has been investigated since the 1960s for its particular magnetic structure and properties^1^. Recently, DFO has drawn renewed attention because of, e.g., its laser or terahertz wave induced spin dynamics^2^,^3^,^4^,^5^,^6^,^7^,^8^, magnetic-field- or uniaxial-stress-induced multiferroic state^9^,^10^,^11^, and Mossbauer spectrum^12^. DFO, like the other rare-earth orthoferrites, is a distorted perovskite crystallizing in an orthorhombic lattice with the Pbnm space group. There are two magnetic sublattices in DFO, namely those associated with Fe- and Dy-ions, which provide three different types of magnetic interactions, namely, Fe^3+^- Fe^3+^, Fe^3+^- Dy^3+^, and Dy^3+^- Dy^3+^ couplings. The Fe^3+^- Fe^3+^ exchange energy determines the predominant magnetic ordering of the iron sublattice below the first Néel temperature (*T*_*N1*_) of about 650 K, that is, a G-type antiferromagnetic structure in which nearest-neighboring irons have anti-parallel spins. In addition, because of a specific Dzyaloshinskii-Moriya (DM) interaction mediated by the tilting of the oxygen octaheda^13^, the Fe spins are not perfectly collinear but, instead, are slightly canted with respect to one another. Indeed, in the so-called Γ_4_ configuration of DFO, which is the most stable magnetic state for the temperature region ranging from *T*_*N1*_ down to 50 K, the G-type antiferromagnetic vector lies along the *a*-axis, and the aforementioned canting provides a weak ferromagnetic component (overt canting) along the *c*-axis, as well as a weak C-type antiferromagnetic component (hidden canting) along the *b*-axis^1^,^14^. At 50 K, a spin reorientation (SR) transition to a so-called Γ_1_ state occurs^15^, in which the G-type antiferromagnetic vector now points along *b*, the overt canting vanishes, and two new hidden cantings of A and C types appear along the *a* and *c*-axes, respectively^1^,^14^,^16^.

The antiferromagnetic transition temperature (*T*_*N2*_) of the Dy^3+^ sublattice is two order of magnitudes lower than *T*_*N1*_, namely about 4.5 K. The resulting Dy^3+^ spin configuration was reported to be G_x_A_y_ in a previous study on DyAlO_3_^17^. Moreover, when a magnetic field is applied along the *c*-axis in this particular state, the system exhibits a magnetic-field-induced phase transition and an electric polarization appears^18^. Furthermore, both weak ferromagnetism and ferroelectricity are also obtained when applying stress along the [110] direction to the low-temperature state with both Dy and Fe spins ordered.

A specific interaction between Fe and Dy neighboring ions can also exist in the Γ_4_(G_x_, A_y_, F_z_) state, and is of the utmost importance since it can result in a net magnetization of Dy^3+^ ions being either parallel or antiparallel to the net Fe^3+^ sublattice moment aligned along the *c*-axis^19^,^20^. Previous magnetizing studies of the Γ_4_ phase of DFO single crystals reported that the magnetizations of Dy and Fe sublattices are parallel to each other^1^,^21^. However, these studies were carried out with a relative large applied field (about 500 Oe)^21^, which may force both the overt canting component of Fe^3+^ sublattice, F_Fe_, and net Dy^3+^ moment component, F_Dy_, to arrange along the field’s direction. Therefore, it is not clear whether such experiments shed light on the intrinsic, zero-field spin configurations and magnetizing behavior of the material.

In order to uncover the basic spin configuration, in general, and reveal the relative arrangement of F_Fe_ and F_Dy_ in the Γ_4_ phase for different fields and temperatures, in particular, we employ here small as well as large applied fields at various temperatures. The present work revisits the pioneering studies of refs 1 and 21, as our results suggest that that F_Fe_ and F_Dy_ tend to align antiparallel to each other, and not parallel as previously proposed. It is also discovered that the application of magnetic fields can result in a magnetic transition, driving F_Dy_ to align parallel to F_Fe_. Importantly, an original temperature-versus-magnetic field phase diagram within the Γ_4_ phase is further provided here, with this diagram exhibiting three different regions, each possessing its own distinct behavior for the dependence of the weak magnetization as a function of the magnetic field (i.e., linear *versus* constant or, alternatively, presenting either behavior depending on the history of the sample). Our results also reveal the precise conditions required to induce the reversal of the magnetization of the Dy sublattice and the flip of the Fe spins. All these (previously overlooked) possibilities for controlling the magnetic state and response of DyFeO_3_ render a highly tunable material, with potential important applications in the context of spintronic devices.

## Results

### Crystal Growth, Sample Preparation and Basic Characterization

**Figure 1:**
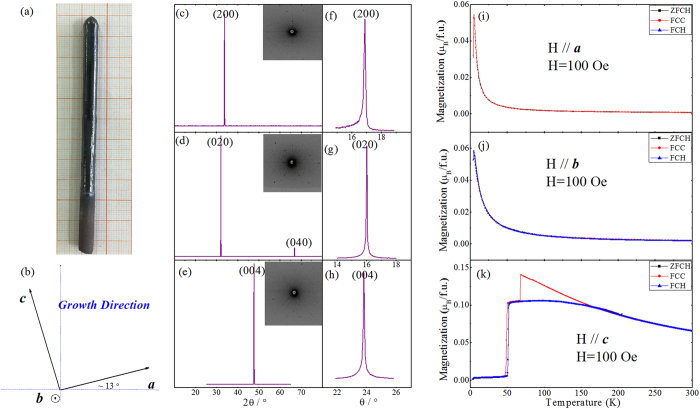
Characteristics and properties of the grown DyFeO_3_ (DFO) single crystal. (**a**) Photograph of it; (**b**) Sketch to display its axes with respect to crystallographic directions; (**c–e**) Its X-ray diffraction (XRD) patterns along the a-, b-, and c-axis, respectively, with the inset displaying the Laue photographs; (**f–h**) Its XRD rocking curves corresponding to (200), (020), and (004) peaks, respectively; (**i–k**) a-, b- and c-axis magnetization, respectively, as a function of temperature, for ZFCH, FCC and FCH (see text) measurements with an applied field of 100 Oe.

Figure 1(a) shows the DFO single crystal grown in floating air, with a 6 mm diameter and a 85 mm length. The as-grown crystal rod presents a pair of parallel cleavage planes along the growth direction, whose normal vector is confirmed to be the *b*-axis by Laue camera. Further orientation results are displayed in Fig. 1(b) by means of a sketch, indicating that the *c*-axis is slightly canted (~13°) from the growth direction. We then cut this crystal rod into a cuboid with a length of 2.4 mm, 2.6 mm and 1.4 mm along the *a*, *b*, and *c* axes, respectively. Figure 1(c–e) shows the X-ray Diffraction (XRD) patterns, which allow to identify characteristic peaks for each axis. Note that the insets of Fig. 1(c–e) are Laue photographs for each crystallography axis corresponding to the XRD peaks. Moreover, Fig. 1(f–h) show the XRD rocking curves for the (200), (020), and (004) peaks, indicating that the full-width at half-maximum (FWHM) is 0.11, 0.05 and 0.08 degree, respectively. Both the XRD and Laue data demonstrate that a high quality DFO single crystal sample was successfully grown under our conditions. Moreover, magnetic properties are displayed in Fig. 1(i–k), and measured via three different procedures – i.e., ZFCH, FCC and FCH–all using an applied field of 100 Oe (see Methods to describe these procedures).

As shown in Fig. 1(i,j), the *a*- and *b*-axis magnetizations, whose ZFCH, FCC and FCH curves all coincide, show a rapid upturn below 50 K, which corresponds to the SR transition from Γ_4_ to Γ_1_. All these magnetizations also exhibit a sharp decrease at *T*_*N2*_ ~ 4.5 K, due to the formation of the antiferromagnetic (G_x_A_y_) state of the Dy spins. The magnetization along the *c*-axis is different from the *a*- and *b*-axis magnetizations. For instance, a large *c*-axis magnetization is present at the highest studied temperatures, due to the weak ferromagnetism (WFM) of the Fe^3+^ sublattice, and an abrupt decrease occurs at 50 K as a result of the SR transition. Further cooling within the Γ_1_ state results in a rather negligible *c*-axis magnetization value. It is important to realize that Fig. 1k shows that our measured c-axis magnetization in the ZFCH procedure (which coincides with the FCH curve) is approximately constant between 50 K and 200 K, which contrasts with previous studies reporting a gradually decrease with temperature above the SR transition^11^,^21^. This difference is likely due to the application of relatively large magnetic fields (well above our used value of 100 Oe) in these previous works. Moreover, another new result is found here, namely, the FCC curve is separated from the two heating curves (ZFCH and FCH); such a behavior is similar to the one observed for the *a*-axis magnetization of dysprosium samarium orthoferrite single crystals^14^.

### The Relative Orientation between Fe and Dy Sublattice Moments

**Figure 2:**
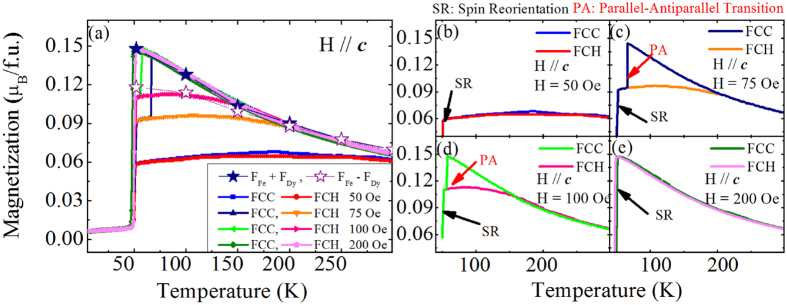
Temperature dependence of the c-axis magnetization for a DFO sample that is initially demagnetized at 500 °C. (**a**) FCC and FCH measurements (solid lines) under different magnetic fields of 50, 75, 100 and 200 Oe, with the star symbols reporting the values of F_Fe_ + F_Dy_ and F_Fe_ − F_Dy_ obtained from Fig. 4. Zoom-in magnetization curves under the magnetic fields of 50, 75, 100 and 200 Oe are shown in (**b–e**), respectively, with the arrows indicating the spin reorientation (SR) and the parallel-antiparallel (PA) transition.

According to previous studies in rare-earth orthoferrites^14^, the nearly temperature-independent behavior of the ZFCH magnetization curve of Fig. 1k between 50 K and 200 K would be representative of an *antiparallel* coupling between the Fe^3+^ sublattice overt canting (F_Fe_) and its induced rare-earth net moment. On the other hand, the sharp increase of the FCC magnetization of Fig. 1k when cooling the system from 200 K to 65 K is characteristic of a net Dy^3+^ moment (F_Dy_) being *parallel*, rather than antiparallel, to F_Fe_, as also consistent with previous studies where relatively large fields were applied^1^,^11^,^21^. The fact that the FCC magnetization exhibits a sudden jump at 65 K, which results in the convergence with the two curves obtained upon heating (ZFCH and FCH), can therefore be thought of as signaling a parallel-to-antiparallel (PA) transition. In other words, the WFM of the Dy sublattice evolves from being parallel to becoming antiparallel to the WFM associated with the Fe ions. Furthermore, Fig. 2(a–e) explore the effect of the magnitude of the applied magnetic field on the *c*-axis magnetization in the FCC and FCH processes. One can see that there is no PA transition for a field of 50 Oe, as evidenced by the fact that the FCC curve is very similar to the ZFCH one for any investigated temperature. F_Dy_ therefore appears to be antiparallel to F_Fe_ both upon cooling and heating for that small field, thus suggesting that the antiparallel magnetic configuration is the most stable one for the Γ_4_ phase of DFO at zero field. On the other hand, a sudden departure of the FCC curves with respect to ZFCH curve does occur for magnetic fields of 75 Oe and 100 Oe; further, the temperature of the magnetization jump that we interpret as a PA transition gets reduced for increasing the magnetic field (e.g., we get 65 K for 75 Oe, and 57 K for 100 Oe). Another interesting feature is that, for an applied field of 200 Oe, the FCH curve does not display any plateau within the stability range of the Γ_4_ phase, but rather follows closely the FCC curve for any temperature. Such a result indicates that, for that larger field, F_Dy_ is always parallel to F_Fe_ both in the cooling and heating processes above the SR critical temperature.

**Figure 3:**
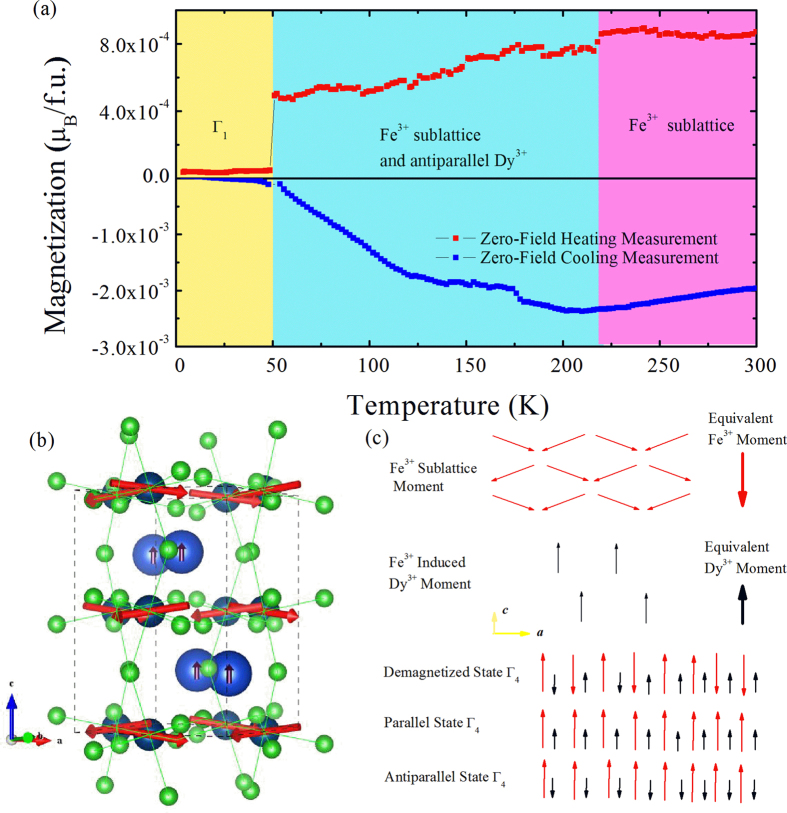
Magnetic characteristics for a DFO sample that is initially demagnetized at 500 °C. (**a**) Temperature behavior of the c-axis magnetization as measured in a quasi-zero-field cooling and heating processes. (**b**) Representation of the spin structure of DFO in the Γ_4_ configuration; small arrows at the Dy cations indicate the weak magnetization induced by the interaction with the Fe spins. (**c**) Schematization of different proposed magnetic states.

To ratify the naturally antiparallel alignment of F_Fe_ and F_Dy_, we conducted another experiment whose results are reported in Fig. 3(a). The demagnetized (please see the details in Methods) DFO was then placed into the chamber, cooling it down to 4 K and then heating it up. The magnetization measurements were performed both during the cooling (Zero-Field Cooling Measurement) and heating (Zero-Field Heating Measurement) processes. As shown in Fig. 3(a), upon cooling we measure a *negative* c-axis magnetization within the stability range of the Γ_4_ phase; for example, a value of 2 × 10^−3^μ_B_/f.u. is found at the highest investigated temperature of 300 K. This magnetization is about thirty times smaller than the one reported in Fig. 1k (which is close to 6 × 10^−2^μ_B_/f.u.). Such small negative value arises from the fact that (i) our thermal demagnetization process favors a vanishing F_Fe_ (as a result of magnetic moments of iron ions being randomly oriented up and down, as schematized in Fig. 3(c), which is a direct consequence of the existence of different antiferromagnetic domains with differently oriented F_Fe_ moments, but (ii) the downward geomagnetic field slightly magnetizes the Fe sublattice, as modeled in Fig. 3(b)). When cooling the system from 300 K to 210 K, the total magnetization remains small and negative but slightly increases in magnitude to reach 2.5 × 10^−3^ μ_B_/f.u., which we attribute to the increasing ordering of the Fe spins. Further cooling below 210 K and down to 50 K results in a total magnetization decreasing in strength until becoming rather small (about 1 × 10^−4^ μ_B_/f.u.). This stationary point at about 210 K likely indicates the appearance of F_Dy_, which is induced by, and antiparallel to, F_Fe_. This net moment of Dy ions, F_Dy_, then grows as the temperature is reduced down to 50 K, which explains the concomitant decrease of the magnitude of the *total* magnetization. The spin reorientation then happens, which yields an almost complete annihilation of the total magnetization below 50 K. Interestingly, Fig. 3(a) further shows that the heating process significantly differs from the cooling one, as evidenced by the fact that the total magnetization upon heating is now *positive* after the Γ_1_ to Γ_4_ SR transition has occurred.

**Figure 4:**
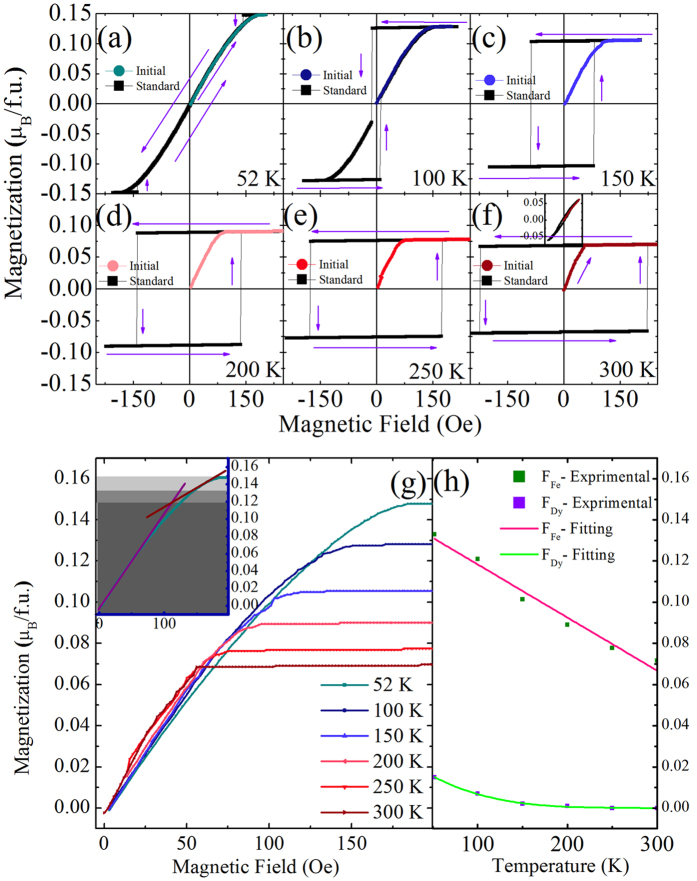
Magnetic properties of a grown DFO sample that is initially demagnetized at each studied temperature. (**a–f**) Magnetization-*versus*-magnetic field hysteresis loops measured at temperatures of 52, 100, 150, 200, 250 and 300 K, respectively. Violet arrows indicate the followed path in terms of the variation of the applied magnetic field, and the inset of Panel (**f**) zooms in the region extending from 0 to 50 Oe. (**g**) The initial part of these loops for the different investigated temperatures. The inset of (**g**) shows the decomposition discussed in the text of this initial part at 52 K, in order to extract F_Fe_ and F_Dy_. (**h**) The temperature behavior of the resulting F_Fe_ and F_Dy_.

Let us now discuss in more detail the magnetic response of DFO in the Γ_4_ phase, to see whether it offers additional hints on the spin structure and interplay between the Fe and Dy sublattices. Figure 4(a–f) display the magnetization hysteresis loops (MH curves) measured at several temperatures in the relevant range. DFO is demagnetized before conducting each MH measurement, in order to get a nearly vanishing initial magnetization. We find that the magnetization increases when applying a magnetic field (starting from the M ~ H ~ 0 point) until reaching a plateau for the largest (positive) applied fields during the MH process. The existence of such a plateau shows that the WFM component of the total magnetization is saturated for these relatively large fields. Note that these saturated values are also consistent with the MT curves reported in Fig. 2(e) for the FCC and FCH measurements corresponding to the application of fields up to 200 Oe.

In Fig. 4(g) we show the *initial part* of the MH curves (i.e., starting from a demagnetized sample and progressively increasing the field’s magnitude) for all studied temperatures. These results indicate that the total magnetization first increases linearly with the field, and then departs from linearity at a certain field magnitude. We can conjecture that the response in the first segment is dominated by the relatively large F_Fe_ canting, while the relatively small F_Dy_ is probably still pointing along the opposite direction. Note that, in this regime, F_Dy_ will be subject to two competing forces that both scale linearly with the applied magnetic field: on one hand we have the direct action of the applied field, which drives both F_Fe_ and F_Dy_ pointing along the field’s direction; on the other hand we have the anti-parallel coupling with F_Fe_, which itself grows linearly with the field. As the applied field grows, F_Fe_ eventually saturates, and F_Dy_ progressively aligns parallel to the applied field (and to F_Fe_) in the second segment of the response.

Following this picture, we quantitatively analyzed the results of Fig. 4(g), in the following way: we first draw a (purple) line fitting the data at smaller fields and then another line fitting data at higher fields (see the inset of Fig. 4(g) for the case of 52 K). The magnetization value associated with the intersection of these two lines is interpreted as being the *difference* between the saturated values of F_Fe_ and F_Dy_, while the plateau of the MT curves in Fig. 4(g) corresponds to the *sum* of the saturated values of F_Fe_ and F_Dy_. Thus, for instance, the inset of Fig. 4(g) shows that the saturated values of F_Fe_ and F_Dy_ at 52 K are 0.133 μ_B_/f.u. and 0.015 μ_B_/f.u., respectively. Such a procedure therefore allows us to extract the saturated values of F_Fe_ and F_Dy_ at *all* investigated temperatures, and the corresponding results are shown in Fig. 4(h). It is interesting to realize that F_Dy_ vanishes above 200 K, as consistent with the results in Figs 1k, 2c,d and 3a. Moreover, the values of F_Fe_ + F_Dy_ and F_Fe_ − F_Dy_ thus obtained are also reported in Fig. 2(a) by means of symbols; we find that the total saturated WFM values are consistent with the FCC MT curves for fields above 75 Oe, while the obtained difference of saturated moments agree well with data of the FCH MT curves for fields close to 100 Oe field. Note that the value of 100 Oe roughly corresponds to the field at which the slope of the MH curve changes (see inset in Fig. 4(g)), supporting the validity of our analysis.

### Mapping of the WFM States

Let us now focus on the hysteresis characteristics of DFO in the Γ_4_ phase, trying to understand the origin of the various behaviors obtained. For the purpose of this discussion, it is convenient to introduce what we denote as the *standard* MH hysteresis loops in Fig. 4(a–f), that is, the loops one obtains by starting from the maximum *positive* field of 250 Oe, then continuously decreasing this field down to a *negative* field of −250 Oe, and finally continuously varying again this field from the aforementioned negative to positive maximum fields to complete the cycle. Interestingly, and as shown in Fig. 4, these standard loops adopt a square-like form for temperatures above 150 K. In particular, once the plateau is reached from the initial state, the MH curves never display a linear behavior again. In contrast, at temperatures below 150 K, we find that it is impossible to obtain the square-loop behavior: by application of a magnetic field we reach a plateau as in the cases above 150 K; yet, when the field decreases and eventually changes sign, at some point the material falls back (abruptly in some cases, like at 100 K in Fig. 4(b)) into a state with a linear MH characteristic, and the transition to the state with large but opposite magnetization is ultimately gradual.

**Figure 5:**
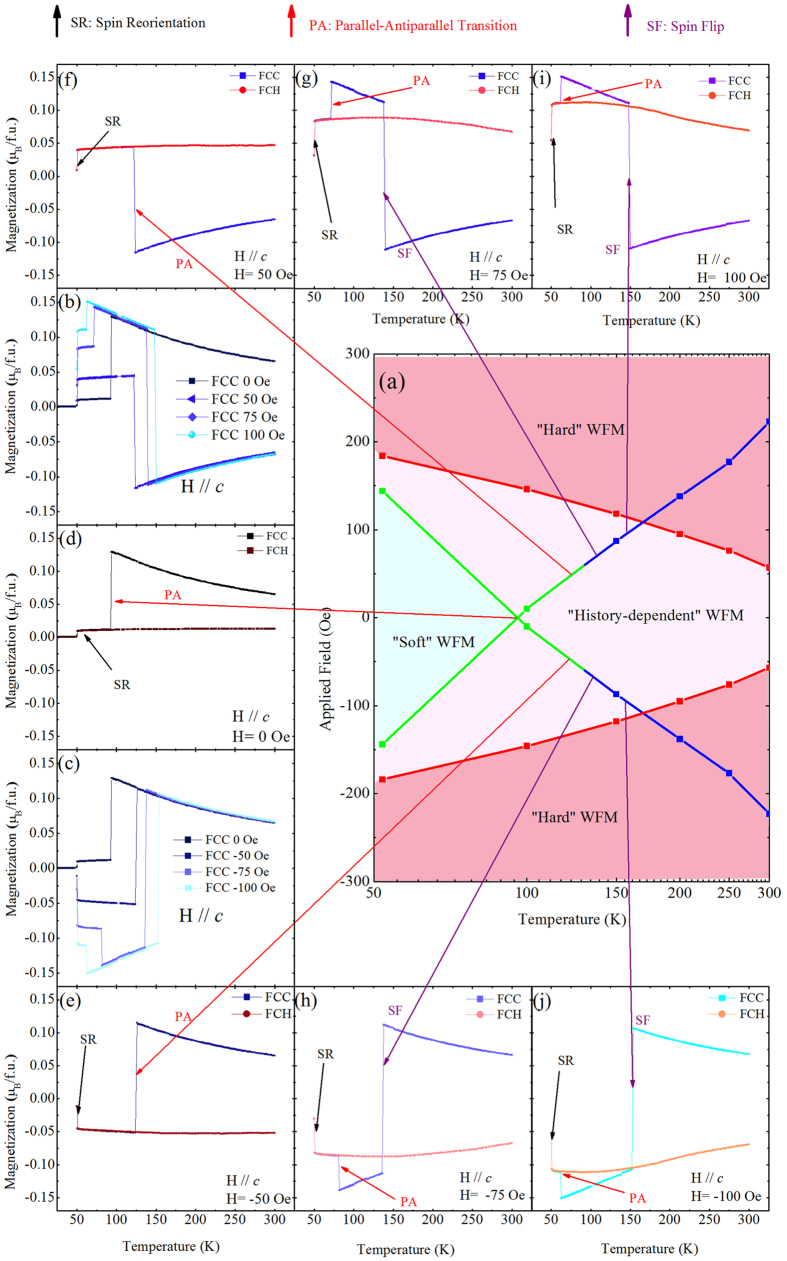
Other magnetic characteristics of DFO. (**a**) Proposed magnetic field vs. temperature phase diagram, involving the “hard”, “history-dependent” and “soft” WFM states as well as transition lines (red for the “history-dependent”/“hard” border, blue for the spin flip (SF) between two “hard” WFM states having opposite magnetization and green for the parallel to antiparallel (PA) transition). The square symbols gather data point obtained from Fig. 4; (**b–j**) FCC c-axis magnetization-versus-temperature under various applied fields for magnetized DFO. FCH measurements are also reported in panels (**d–j**). The results of panels (**b–j**) allow to complete and confirm the proposed, original phase diagram of panel a.

To rationalize these behaviors, it is useful to distinguish three regions in the field-temperature phase diagram of the material, as shown in Fig. 5(a): We have regions in which the material always presents a flat MH characteristic, a situation that we denote “hard WFM” state. The boundary of this region is given by the temperature-dependent magnetic field values at which the MH plateau is reached (see Fig. 4); this boundary is marked by red lines and squares in Fig. 5(a). Then, we have a region of the phase diagram in which the material can present either a square-like MH curve or a linear one, depending on the history of the sample. This is what we call the “history-dependent WFM” region in Fig. 5(a). Finally, there is a region of the phase diagram within which the measured MH curves are always linear, independently of the history of the sample; this is what we call “soft WFM” region in Fig. 5(a). Such “soft WFM” MH behavior can only be found in low temperature region. Indeed, as can be seen in Fig. 4 and is reflected in the phase diagram of Fig. 5(a), for high enough temperatures (roughly above 90 K) we always obtain square MH characteristics even at zero field; in such conditions, the material can only be in the “history-dependent” or “hard” states, never in the “soft” one.

Moreover, for temperatures above 150 K, Fig. 4(c–f) further show that the magnetization within our standard hysteresis loops abruptly switches from positive to negative values, with the magnitude of this magnetization being conserved, when under a magnetic field. For instance, such a switching occurs for a magnetic field of −225 Oe at 300 K. These jumps are reported by means of blue squares in the magnetic field versus temperature phase diagram depicted in Fig. 5(a), and correspond to a transition between two “hard” WFM states having opposite long-range-ordered F_Fe_–since F_Dy_ is rather small above 150 K as shown in Fig. 4(h).

It is also interesting to realize that, for T = 100 K, the standard hysteresis loop switches from a positive value of the magnetization equal to 0.13 μ_B_/f.u (which corresponds to the F_Fe_ + F_Dy_ sum one can extract from Fig. 4(h) at T = 100 K, and as characterizing a “hard” WFM state) to a negative one equal to −0.03 μ_B_/f.u (as representative of a “soft” WFM state, since the magnetization then linearly varies with H), for an applied magnetic field of about −10 Oe. Interestingly, this negative magnetization of −0.03 μ_B_/f.u is neither equal to the value of -(F_Fe_ + F_Dy_) = −0.128 μ_B_/f.u nor to the value of -(F_Fe_ − F_Dy_) = −0.113 μ_B_/f.u one can deduce from Fig. 4(h). Similarly, the jump between the “hard” and “soft” states for magnetic field close to 140 Oe at T = 52 K does not correspond to a change of magnetization from (F_Fe_ + F_Dy_) = 0.147 μ_B_/f.u to (F_Fe_ − F_Dy_) = 0.117 μ_B_/f.u one can obtain from Fig. 4(h). To explain such results, it is reasonable to assume that “soft” and even “hard” WFM states, *at and below 100* *K*, are formed by domains having opposite directions for the antiferromagnetic (AFM) vector of the Fe sublattice. When no field is applied and the total magnetization is vanishing (e.g., at the beginning of the initial hysteresis loop for T = 100 K and 52 K, but also during the standard hysteresis loop at T = 52 K, see Fig. 4(a,b)), these AFM domains should also possess opposite directions for the magnetization of the Fe ions (positive or negative along the c-axis), since, within the Fe sublattice, the direction of the G-type antiferromagnetic vector dictates the direction of the WFM via a Dzyaloshinskii-Moriya (DM) interaction^11^. Moreover, these antiferromagnetic domains should also naturally favor opposite directions for the magnetization of the Dy ions (negative or negative along the c-axis), since F_Dy_ and F_Fe_ likely energetically prefer to be antiparallel to each other via another DM interaction^19^. However, at T = 100 K and H = −10 Oe during the standard hysteresis loop, the resulting “history-dependent” WFM state has a negative, small, but non-zero total magnetization, which can be explained by the facts that (i) there is a competition between the magnetic field desiring to make *all* the weak magnetizations of the Fe and Dy ions (i.e., belonging to the different AFM domains) aligned along its direction, and the aforementioned DM interactions^11^,^19^ that prefer to induce the WFM of the Fe ions and/or the WFM of the Dy ions to be aligned opposite to the applied magnetic field in some AFM domains; and (ii) the magnetic field wins more and more this competition as its strength grows. Note that items (i) and (ii) can also imply that the “hard” WFM state is formed by AFM domains below 100 K too, but with the WFMs of the Fe and Dy ions now completely following the magnetic field (i.e., not anymore reacting to the DM interactions^11^,^19^) and therefore saturating in value.

The analysis of the results of Fig. 4 therefore supports the idea that “soft” and even “hard” WFM states existing below 100 K can possess AFM domains, for which the ratio between domains with up magnetization and domains with down magnetization can be controlled by the magnitude and sign of the applied magnetic field. Note that, on the other hand, the “hard” WFM states above 150 K may be single monodomain states, which would explain the fact that the standard hysteresis loop has a perfect square shape and, therefore, that “soft” WFM states do not appear anymore in this standard loop.

Note that the facts that it is possible to access the antiferromagnetic-monodomain at relatively high temperatures, while it becomes essentially impossible (for the applied magnetic fields) at temperatures of 100 K and below, are actually the behaviors to expect. As a matter of fact, achieving the transformation to a monodomain state should be easier (i.e., it should require smaller applied fields) when the antiferromagnetic order is not strongly developed and/or the material has enough thermal energy, i.e., at higher temperatures.

Moreover and in order to complete the phase diagram of Fig. 5a, we decided to perform additional experiments, that consist in measuring the *c*-axis magnetization-versus-temperature curves of a DFO sample that is first magnetized at 300 K by a positive field along the c-axis of 200 Oe (respectively, negative field of −200 Oe) and then cooled down to 4 K and then heated up to 300 K under negative fields of −50, −75 or −100 Oe (respectively, positive fields of +50, +75 and + 100 Oe). The resulting FCC and FCH MT functions are displayed in Fig. 5(b, c, e–j). For the sake of completeness and for comparison, we also conducted similar measurements but for which no magnetic field is applied during cooling and heating after the DFO sample has been magnetized under a field of 200 Oe (see results in Fig. 5(d)). Note that this initial application of a field of 200 Oe generates a total magnetization of 0.07_B_/f.u. at 300 K (that is similar to the saturated value shown in Fig. 4f), and therefore results in the formation of a “hard” WFM state.

One can see that for fields equal to or above 75 Oe in magnitude, the total FCC magnetization can suddenly switch and nearly completely reverts its value, via a first jump, for a specific field-dependent temperature that is always larger than 130 K. Such data allow us to extend the blue lines of Fig. 5(a), and correspond to the spin flip (SF) transition between two “hard” WFMs states having opposite and long-range-ordered F_Fe_ magnetization. Moreover, for these fields equal to or above 75 Oe in magnitude, a second type of jump of the FCC occurs at smaller temperature (e.g., equal to 72 K for H = 75 Oe), below which the FCC and FCH curves become identical. Unlike the first type of jump and as evidenced in Fig. 5(b,f), this second type of jump is also happening for fields of 50 Oe and even 0 Oe magnitude and corresponds to the aforementioned PA transition below which F_Dy_ naturally prefers to be antiparallel (rather than parallel) to F_Fe_. This second type of jumps therefore allows us to add more point to the green curve of Fig. 5(a), which is associated with the transition from “hard” WFM to “soft”/“history-dependent” WFM. Interestingly, the continuity one can see in Fig. 5a between the data extracted from Fig. 4 and those obtained from Fig. 5(b–j) demonstrates that the phase diagram of Fig. 5(a) is universal, in the sense that it applies to MH measurements done at fixed temperature (as in Fig. 4) but also to MT experiments conducted at fixed magnetic field (as in Fig. 5).

## Discussion

Weak ferromagnetism has been systematically investigated in a high-quality single crystal of DFO grown by an optical floating zone method. Magnetic measurements reveal that the Γ_4_ magnetic state naturally prefers to present F_Fe_ and F_Dy_ moments being antiparallel to each other. We further found that relatively small magnetic fields can transform the antiparallel coupling into a parallel one, with the resulting state being coined “hard” WFM and for which the magnetization is nearly independent from the applied field. A magnetic field-*versus*-temperature phase diagram is constructed, mapping the “hard” WFM and “soft” WFM states but also a third state denoted as “history-dependent” WFM which can either be “hard” or “soft” depending on the history of the procedure used. This phase diagram also precisely predicts the sudden jumps of different natures (e.g., between two “hard” WFM states of opposite magnetization or between “soft” and “hard” WFM states), which is of obvious importance for devices exploiting spin switching phenomena.

## Methods

A single crystal DFO was grown by an optical-floating-zone method (Crystal System Inc., type FZ-T-10000-H-VI-P-SH). The compounds of feed and seed rods were prepared by the solid state reaction of the raw materials Dy_2_O_3_(99.9%), and Fe_2_O_3_(99.99%) with the proper cation stoichiometry, which was calculated by the target compound. During the growth process, the molten zone moved upwards at a rate of 3 mm/h, with the seed rod (lower shaft) and the feed rod (upper shaft) counter rotating at 30 rpm in flowing air. Employing an X-ray Laue photograph (Try-SE. Co, Ltd.), we determined the crystallographic orientations, and cut the crystal into a 2.4 × 2.6 × 1.4 mm^3^ sample. The orientations were further verified by means of θ-2θ linear scans, using a high-resolutionX-ray diffraction (Bruker D8 Discover with 4-bounce Ge(220) monochromator and Cu K_α1_ radiation (λ = 1.5406 Å)). The X-ray diffraction rocking curve on the (200), (020) and (004) diffraction peaks were also taken using this equipment. Measurements of the magnetization as a function of temperature and magnetic field were carried out using a Physical Property Measurement System (PPMS-9, Quantum Design) and a superconducting quantum interference device (SQUID) magnetometer (MPMS XL-5, Quantum Design), respectively, with the direction of the applied magnetic field being parallel to the corresponding crystallography axis. Different procedures were employed to acquire the temperature dependence of the magnetization (MT). For instance, we cooled the sample under no field down to 4 K, and then applied a magnetic field while heating the system; MT measurements were obtained during this latter heating process, which we denote as zero-field-cooling heating (ZFCH). We also cooled the system down to 4 K but now under an applied magnetic field, and measured the resulting MT curve during that process, which is coined field-cooling cooling (FCC) measurements. Finally, at the end of the FCC process, we also heated the system under the same magnetic field and measured the magnetization as a function of temperature. These measurements are denoted here as field-cooling heating (FCH). Furthermore, the magnetization *vs*. magnetic field isotherm curves were measured at several representative temperatures by changing the field at these temperatures.

Note that before all of those measurements, both the sample and the MPMS chamber are pretreated: (1) the sample was demagnetized at 500 °C, which is higher than the *T*_*N*1_ to disorder the orientation of WFM components; (2) a Pd standard sample, which is commonly used in MPMS to perform a precise small field correction before the measurements, is employed to obtain a quasi-zero-field measurements chamber.

## Additional Information

**How to cite this article**: Cao, S. *et al*. Tuning the Weak Ferromagnetic States in Dysprosium Orthoferrite. *Sci. Rep*. **6**, 37529; doi: 10.1038/srep37529 (2016).

**Publisher’s note:** Springer Nature remains neutral with regard to jurisdictional claims in published maps and institutional affiliations.

## References

[b1] White R. L. Review of recent work on the magnetic and spectroscopic properties of the rare-earth orthoferrites. Journal of Applied Physics 40, 1061–1069 (1969).

[b2] Afanasiev D., Zvezdin A. K. & Kimel A. V. Laser-induced shift of the Morin point in antiferromagnetic DyFeO_3_. Optics Express 23, 23978–23984 (2015). 26368488 10.1364/OE.23.023978

[b3] Iida R. . Spectral dependence of photoinduced spin precession in DyFeO_3_. Physical Review B 84, 064402 (2011).

[b4] Mikhaylovskiy R. V. . Terahertz magnetization dynamics induced by femtosecond resonant pumping of Dy^3+^ subsystem in the multisublattice antiferromagnet DyFeO_3_. Physical Review B 92, 094437 (2015).

[b5] Reid A. H. M., Rasing T., Pisarev R. V., Duerr H. A. & Hoffmann M. C. Terahertz-driven magnetism dynamics in the orthoferrite DyFeO_3_. Applied Physics Letters 106, 082403 (2015).

[b6] Yamaguchi K., Kurihara T., Watanabe H., Nakajima M. & Suemoto T. Dynamics of photoinduced change of magnetoanisotropy parameter in orthoferrites probed with terahertz excited coherent spin precession. Physical Review B 92, 064404 (2015).

[b7] Afanasiev D. . Control of the Ultrafast Photoinduced Magnetization across the Morin Transition in DyFeO_3_. Physical Review Letters 116, 097401 (2016). 26991201 10.1103/PhysRevLett.116.097401

[b8] Kimel A. V. . Ultrafast non-thermal control of magnetization by instantaneous photomagnetic pulses. Nature 435, 655–657 (2005). 15917826 10.1038/nature03564

[b9] Nakajima T., Tokunaga Y., Taguchi Y., Tokura Y. & Arima T.-h. Piezomagnetoelectric Effect of Spin Origin in Dysprosium Orthoferrite. Physical Review Letters 115, 197205 (2015). 26588412 10.1103/PhysRevLett.115.197205

[b10] Stroppa A., Marsman M., Kresse G. & Picozzi S. The multiferroic phase of DyFeO_3_: an ab initio study. New Journal of Physics 12, 093026 (2010).

[b11] Tokunaga Y., Iguchi S., Arima T. & Tokura Y. Magnetic-field-induced ferroelectric state in DyFeO_3_. Physical Review Letters 101, 097025 (2008). 10.1103/PhysRevLett.101.097205 18851654

[b12] Reddy S. S. K. . Structural, electrical, magnetic and Fe-57 Mossbauer study of polycrystalline multiferroic DyFeO_3_. Journal of Magnetism and Magnetic Materials 396, 214–218 (2015).

[b13] Bellaiche L., Gui Z. & Kornev I. A. A simple law governing coupled magnetic orders in perovskites. Journal of Physics-Condensed Matter 24, 312201 (2012). 10.1088/0953-8984/24/31/312201 22776811

[b14] Zhao W. . Spin reorientation transition in dysprosium-samarium orthoferrite single crystals. Physical Review B 91, 104425 (2015).

[b15] Yamaguchi T. Theory of spin reorientation in rare-earth orthochromites and orthoferrites. Journal of the Physics and Chemistry of Solids 35, 479–500 (1974).

[b16] Prelorendjo L. A., Johnson C. E., Thomas M. F. & Wanklyn B. M. Spin reorientation transitions in DyFeO_3_ induced by magnetic fields. Journal of Physics C: Solid State Physics 13, 2567 (1980).

[b17] Holmes L. M., Van Uitert L. G., Hecker R. R. & Hull G. W. Magnetic Behavior of Metamagnetic DyAlO_3_. Physical Review B 5, 138–146 (1972).

[b18] Wang J. . Simultaneous occurrence of multiferroism and short-range magnetic order in DyFeO_3_. Physical Review B 93 (2016).

[b19] Zhao H. J., Íñiguez J., Chen X. M. & Bellaiche L. Origin of the magnetization and compensation temperature in rare-earth orthoferrites and orthochromates. Physical Review B 93, 014417 (2016).

[b20] Zou Y. H. . Spin dependent electrical abnormal in TbFeO_3_. Journal of Alloys and Compounds 519, 82–84 (2012).

[b21] Zhao Z. Y. . Ground state and magnetic phase transitions of orthoferrite DyFeO_3_. Physical Review B 89, 224405 (2014).

